# Vowel decoding from single‐trial speech‐evoked electrophysiological responses: A feature‐based machine learning approach

**DOI:** 10.1002/brb3.665

**Published:** 2017-04-26

**Authors:** Han G. Yi, Zilong Xie, Rachel Reetzke, Alexandros G. Dimakis, Bharath Chandrasekaran

**Affiliations:** ^1^ Department of Communication Sciences & DisordersMoody College of CommunicationThe University of Texas at AustinAustinTXUSA; ^2^ Department of Electrical and Computer EngineeringCockrell School of EngineeringThe University of Texas at AustinAustinTXUSA; ^3^ Department of PsychologyCollege of Liberal ArtsThe University of Texas at AustinAustinTXUSA; ^4^ Department of LinguisticsCollege of Liberal ArtsThe University of Texas at AustinAustinTXUSA; ^5^ Institute of Mental Health ResearchCollege of Liberal ArtsThe University of Texas at AustinAustinTXUSA; ^6^ Institute for NeuroscienceCollege of Liberal ArtsThe University of Texas at AustinAustinTXUSA

**Keywords:** EEG, frequency‐following responses, speech decoding, vowels

## Abstract

**Introduction:**

Scalp‐recorded electrophysiological responses to complex, periodic auditory signals reflect phase‐locked activity from neural ensembles within the auditory system. These responses, referred to as frequency‐following responses (FFRs), have been widely utilized to index typical and atypical representation of speech signals in the auditory system. One of the major limitations in FFR is the low signal‐to‐noise ratio at the level of single trials. For this reason, the analysis relies on averaging across thousands of trials. The ability to examine the quality of single‐trial FFRs will allow investigation of trial‐by‐trial dynamics of the FFR, which has been impossible due to the averaging approach.

**Methods:**

In a novel, data‐driven approach, we used machine learning principles to decode information related to the speech signal from single trial FFRs. FFRs were collected from participants while they listened to two vowels produced by two speakers. Scalp‐recorded electrophysiological responses were projected onto a low‐dimensional spectral feature space independently derived from the same two vowels produced by 40 speakers, which were not presented to the participants. A novel supervised machine learning classifier was trained to discriminate vowel tokens on a subset of FFRs from each participant, and tested on the remaining subset.

**Results:**

We demonstrate reliable decoding of speech signals at the level of single‐trials by decomposing the raw FFR based on information‐bearing spectral features in the speech signal that were independently derived.

**Conclusions:**

Taken together, the ability to extract interpretable features at the level of single‐trials in a data‐driven manner offers unchartered possibilities in the noninvasive assessment of human auditory function.

## 1 Introduction

Scalp‐recorded electrophysiological responses to complex auditory signals closely resemble the acoustic properties of the stimuli. These responses are referred to as the frequency‐following response (FFR). Previous studies suggest that the FFR reflects phase‐locked activity from neural ensembles within the auditory system (Gardi, Merzenich, & McKean, 1979; Møller & Jannetta, 1982; Smith, Marsh, & Brown, 1975). Spectral features of the speech‐evoked FFR contain sufficient information reflecting stimulus features, such as the identity of a steady‐state vowel token (Kraus & Nicol, 2005; Krishnan, 1999, 2002). The fidelity of the FFR to the speech stimuli has been associated with individual variability in auditory function in typical (Krishnan, Swaminathan, & Gandour, 2009; Krizman, Marian, Shook, Skoe, & Kraus, 2012; Krizman, Skoe, Marian, & Kraus, 2014; Song, Skoe, Wong, & Kraus, 2008; Wong, Skoe, Russo, Dees, & Kraus, 2007) and atypical/clinical populations, such as those with language and reading disorders (Banai, Abrams, & Kraus, 2007; Chandrasekaran, Hornickel, Skoe, Nicol, & Kraus, 2009; Cunningham, Nicol, Zecker, Bradlow, & Kraus, 2001; Hornickel, Skoe, Nicol, Zecker, & Kraus, 2009; Russo et al., 2008). The FFR as a metric has thus been widely regarded as a potent biomarker of auditory processing (Johnson, Nicol, & Kraus, 2005; Kraus & Chandrasekaran, 2010; Skoe & Kraus, 2010).

A major limitation of the scalp‐recorded FFR is the low signal‐to‐noise ratio at the level of single trials. The FFR is posited to originate from deep structures within the ascending auditory system (Gardi et al., 1979; Møller & Jannetta, 1982; Smith et al., 1975), with possible cortical sources contributing to the lower spectral components (Coffey, Herholz, Chepesiuk, Baillet, & Zatorre, 2016). Therefore, the current standard for FFR signal averaging requires the collection of several thousand trials (Skoe & Kraus, 2010), which precludes the possibility of examining the properties of the FFR at the level of single trials. This limitation constrains FFR research on two fronts. First, the proposed generator of the FFR, the auditory midbrain, exhibits rapid neural adaptation to repeated stimuli (Anderson & Malmierca, 2013; Pérez‐González, Hernández, Covey, & Malmierca, 2012; Malone & Semple, 2001; Zhao, Liu, Shen, Feng, & Hong, 2011). An averaged signal, especially in the context of passive listening, is thus likely to be an aggregate of multiple responses that have undergone adaptation due to the lack of novelty in the incoming acoustic stream. Second, the averaging approach renders it difficult to assess the effects of flexible cognitive demands, such as different attention conditions across trials (Varghese, Bharadwaj, & Shinn‐Cunningham, 2015). The ability to analyze the FFR at the level of single trials could address these limitations, hence enhancing the utility of the responses.

One of the ways in which meaningful information can be extracted from the single‐trial FFR is via machine learning. Machine learning approaches have previously been used to decode phonemes from cortical electrophysiological responses (Hausfeld, De Martino, Bonte, & Formisano, 2012; Mesgarani, Cheung, Johnson, & Chang, 2014; Pei, Barbour, Leuthardt, & Schalk, 2011). Direct electrocorticography methods have been used to show that the firing patterns of cortical neurons can be used to reliably discriminate English phonemes (Mesgarani et al., 2014; Pei et al., 2011), which has been replicated with a noninvasive approach of recording cortical activity (Hausfeld et al., 2012). Unlike their cortical counterparts, the FFR closely mimics the spectrotemporal properties of the original auditory stimuli (Bidelman, 2014), to the degree that listeners can recognize words from the neural responses that have been converted into sound stimuli (Galbraith, Arbagey, Branski, Comerci, & Rector, 1995). Thus, it is conceivable that phonemic decoding would be feasible from the FFRs. Indeed, once the responses are averaged across multiple trials, vowels can be decoded from the FFRs (Sadeghian, Dajani, & Chan, 2015). However, this approach still relies on averaging across hundreds of trials, limiting experimenters’ ability to characterize intersubject variability on a trial‐by‐trial basis.

Here, we used a novel machine learning approach to decode vowels from single trial, speech‐evoked FFRs. We focused on the spectral features observable in the FFR to the vowel sounds, and examine the extent to which vowel related features could be used to classify the stimuli. FFRs were collected from listeners as they passively listened to multiple repetitions (1,000+) of four speech stimuli: two English vowels ([æ] and [u]) produced by two male native English speakers (Hillenbrand, Getty, Clark, & Wheeler, 1995). Each single‐trial FFR was converted into a spectrum, and then projected to a spectral feature space independently derived from the same two vowels ([æ] and [u]) produced by 40 male speakers (Hillenbrand et al., 1995). Finally, we trained a Gradient Boosted Decision Tree model (XGBoost; Chen & Guestrin, 2016) as our classifier. Boosted Decision trees offer state of the art learning performance and also offer high feature interpretability. In this study, we asked the following questions: (1) How well can the individual stimuli (*N* = 4) and vowel tokens (*N* = 2) be decoded from single trials? (2) How many trials are necessary for reliable decoding performance? (3) Are the features used in decoding interpretable? To anticipate, our results, discussed in detail below, showed that the speech tokens can be reliably decoded from single trial FFRs, even with training sets consisting of 50 trials per each stimulus. Furthermore, the spectral feature used to successfully classify the vowels closely corresponded to formant structure of the stimuli. Thus, we demonstrate that phonological information can be extracted from *single‐trial* FFR using a machine learning approach based on interpretable spectral features.

## 2 Materials and Methods

### 2.1 Participants

Young adults (*N* = 38; 30 females; ages 18–35; mean age = 21.6, *SD* = 3.5) were recruited for a large‐scale multi‐session research project from the greater Austin community. A subset of these participants attended an FFR recording session (*N* = 25; 20 females; ages 18–32; mean age = 22.2, *SD* = 4.1), comprising the dataset reported in this study. All participants were native speakers of English, according to an abridged form of LEAP‐Q (Marian, Blumenfeld, & Kaushanskaya, 2007). All participants underwent audiological screening using pure‐tone audiometry and exhibited hearing thresholds of less than 25 dB hearing level at frequencies between 250 and 8,000 Hz (octave steps). Potential participants were excluded if they reported a history of neurological or psychological disorders or ongoing intake of psychogenic medications. All participants were monetarily compensated. All materials and methods were approved by the Institutional Review Board of the University of Texas at Austin. All participants provided written informed consent before their participation in this study.

### 2.2 Construction of the spectral feature space

Nuclei of [æ] and [u] vowels produced by 40 male native English speakers (not presented to the participants) were resampled at the rate of 25 kHz (Hillenbrand et al., 1995). Each of the 80 sounds were converted into a spectrum with a spectral step size of 4 Hz, and truncated between 0 and 4 kHz, leaving 1,000 spectral sampling points. A principal component analysis (PCA) was conducted to calculate a set of non‐covarying principal spectral components that explain the variance across the log‐transformed spectra (Pedregosa et al., 2011). The top 12 components together accounted for 80% of the variance.

### 2.3 Electrophysiological recording procedures

**Figure 1:**
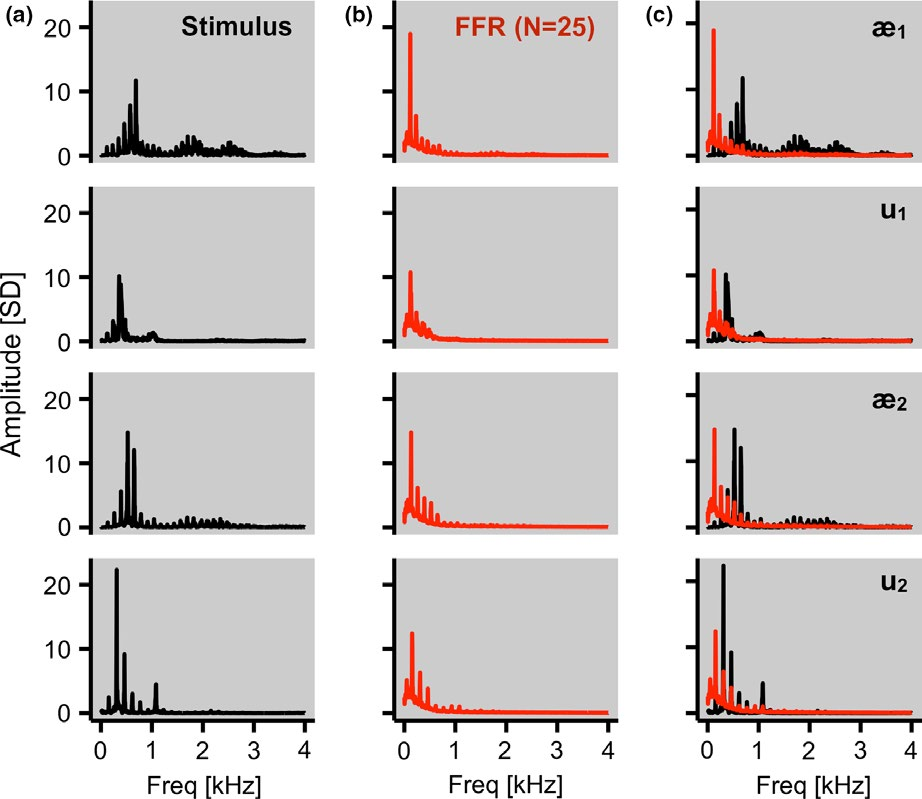
(a) Spectra for [æ] and [u] vowels produced by two male native speakers of English. The *x*‐axis codes frequency ranging from 0 to 4 kHz, in 4‐Hz steps. The *y*‐axis codes relative amplitude at each spectral bin, which has been scaled by the standard deviation of each of the four sound files. (b) Spectra for the frequency following responses collected from 25 participants, which were averaged across 1,000 trials. The *x*‐ and *y*‐axes are identical to those used in (a). (c) Overlaying the two sets of spectra reveals spectral similarity across the stimuli and the responses within each speech token

Electrophysiological responses were recorded using an active Ag–AgCl scalp electrode placed on the Cz site based on the 10–20 system, with an electrode placed on the left mastoid serving as the ground and on the right mastoid as the reference. Impedances for all the electrodes were less than 5 kΩ. During the recording session, participants sat in an acoustically shielded chamber and watched a silent movie of their choice with English subtitles. The stimuli were binaurally presented via insert earphones (ER‐3; Etymotic Research, Elk Grove Village, IL, USA). Participants were instructed to ignore the sounds and focus on the movie. The stimuli were [æ] and [u] vowels produced by two male native English speakers (Figure 1a), which were not used in the construction of the spectral feature space (Hillenbrand et al., 1995), from which the vowel nuclei were extracted using the documented start and end time points, and duration normalized to 250 ms and RMS amplitude normalized to 70 dB sound pressure level. Sounds were presented binaurally at a variable interstimulus interval from 122 to 148 ms. The responses were collected at the sampling rate of 25 kHz using BrainVision PyCorder (1.0.7; Brain Products, Gilching, Germany). Responses to all four stimuli were collected during a single session. The order of stimulus presentation was counterbalanced across participants.

### 2.4 Preprocessing of the frequency‐following responses

After the recording, the responses were preprocessed with BrainVision Analyzer (2.0; Brain Products). First, responses were off‐line bandpass filtered from 80 to 3,500 Hz (12 dB/octave, zero phase‐shift; Aiken & Picton, 2008; Bidelman, Moreno, & Alain, 2013; Krishnan, 2002). Responses were then segmented into epochs of 310 ms (−40 ms before stimulus onset and 270 ms after stimulus onset). Time points were adjusted by 7 ms to account for the neural lag inherent in FFR. After baseline correcting each response to the mean voltage of the noise floor (−40 to 0 ms), trials with activity exceeding the range of ±35 μV were rejected. For each stimulus, at least 1,000 artifact‐free trials were obtained, discarding any additional trials that might have been collected (Skoe & Kraus, 2013).

### 2.5 Projection of the single‐trial FFRs onto the spectral feature space

**Figure 2:**
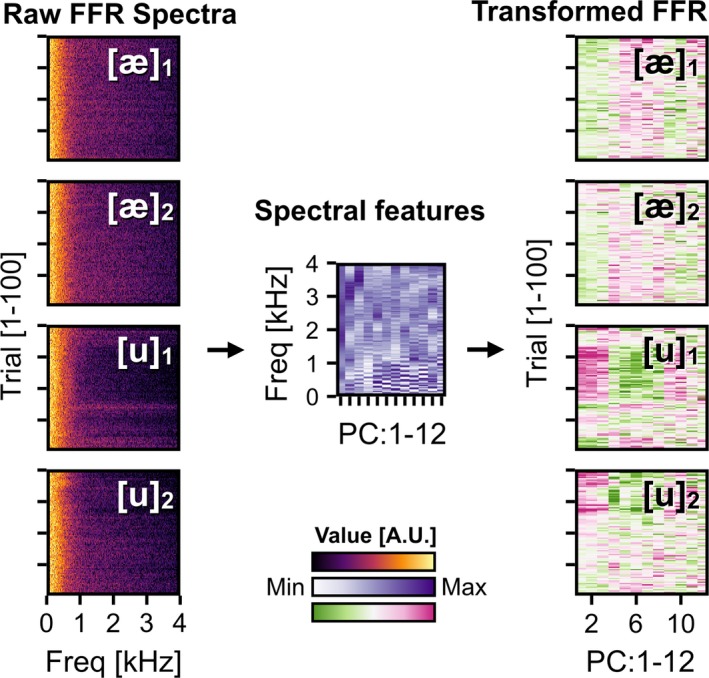
Spectral projection of the single‐trial frequency following responses (FFRs) onto the spectral feature space. Figures are derived from a representative participant. The raw FFR spectra (left) were multiplied by a matrix of 12 vectors (center) corresponding to the top principal components independently derived from spectra of [æ] and [u] vowels produced by 40 male native speakers. This procedure resulted in projection of the raw FFRs onto the 12‐dimensional spectral feature space (right)

The 250‐ms stimulus portion of each of the single‐trial waveforms was converted into a spectrum through steps and parameters identical with those for the construction of the spectral feature space as discussed above (Figure 1b). Grand average spectra of the responses showed a close resemblance to the original stimuli (Figure 1c; Kraus & Nicol, 2005; Krishnan, 1999, 2002; Skoe & Kraus, 2013). Next, the spectra were projected onto the aforementioned 12‐dimensional spectral feature space. To do so, each of the spectra was multiplied by the first two columns of the transformation matrix derived from the PCA performed on the 80 sounds. Therefore, each single‐trial FFR was now represented as a vector of 12 numerical values, which corresponded to the weighting of 12 spectral features (Figure 2).

### 2.6 Decoding the FFRs using a machine learning approach

**Figure 3:**
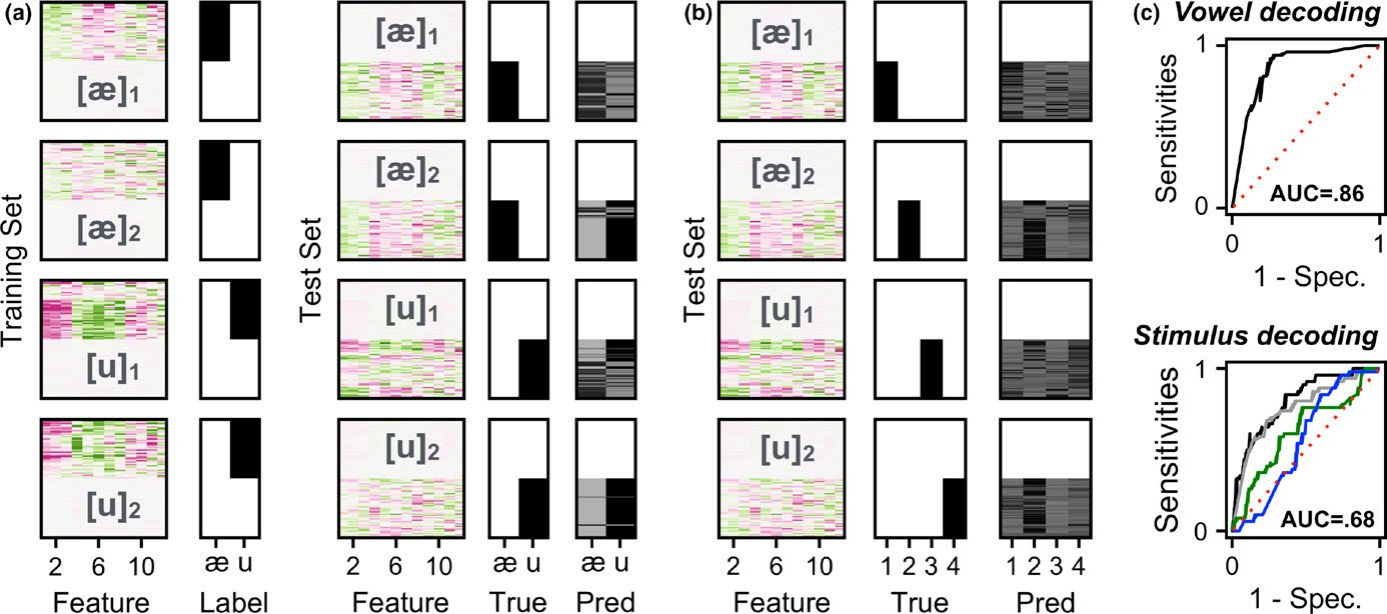
(a) Training‐test scheme for vowel (*N* = 2) decoding. For each participant, a classifier was trained to identify the [æ] and [u] labels from each trial, based on the 12 spectral features. Then, the trained classifier was tested on an independent subset. The resulting prediction vector included values pertaining to the probability of each vowel. In this particular example from a representative participant, the classifier outputs reasonably accurate responses for [æ]_1_ and [u]_2_, but not for [æ]_2_ and [u]_1_. (b) Training‐test scheme for stimulus (*N* = 4) decoding. For each participant, a classifier was trained to identify for [æ]_1_, [æ]_2_, and [u]_1_, and [u]_2_ labels from each trial, based on the 12 spectral features. Then, the trained classifier was tested on an independent subset. The resulting prediction vector included values pertaining to the probability of each of the four stimuli. In this particular example from a representative participant, the classifier outputs reasonably accurate responses for [æ]_1_ and [æ]_2_, but not for [u]_1_ and [u]_2_. (c) Based on the aforementioned probability vectors, a receiver operating characteristics (ROC) curve was generated. The area under the curve (AUC) measure served as a metric of decoding performance. Note that for stimulus decoding, the ROC curve was constructed separately for each stimulus per a one‐versus‐all scheme

Machine learning was conducted on a participant‐by‐participant basis. Each trial was defined as a vector of 12 elements, as calculated from the projection onto the spectral feature space. First, we examined the degree to which vowel tokens (*N* = 2) or individual stimuli (*N* = 4) could be decoded from the single‐trial FFR. To this end, the training set was from the first 950 trials per each stimulus from each participant. A classifier was trained to classify either the vowel ([æ] or [u]; Figure 3a) or stimulus ([æ]_1_, [u]_1_, [æ]_2_, or [u]_2_; Figure 3b) label from the training set.

A gradient boosted decision tree model was initialized with logistic binary classification as the objective, in the case of two‐category classification (i.e., vowels), and with multiclass probability as the objective, in the case of four‐category classification (i.e., individual stimuli) (Chen & Guestrin, 2016). An exhaustive grid search across two hyper‐parameters, number of estimators (from 2 to 12; step size of 2) and maximum depth of each tree (from 2 to 6; step size of 2), was performed based on maximizing the decoding performance within the training set. Then, the performance of the classifier was tested on the remaining test set of 50 trials per each stimulus, separately for each participant. Decoding performance of the classifier was quantified by extracting a receiver operating characteristics curve based on the probability vector of the predicted labels, and calculating the area under each curve (AUC), on a participant by participant basis (Figure 3c). In the case of the stimulus classification, the AUC was calculated for each stimulus via a one‐versus‐all scheme, and then averaged across the stimuli (Galar, Fernández, Barrenechea, Bustince, & Herrera, 2011). This approach was used to circumvent inflation of accuracies due to a response bias in the classifier. Next, we examined the number of total trials necessary for reliable decoding. To this end, the aforementioned decoding procedures were replicated, while varying the size of the training set from 50 to 950 trials per stimulus, with a step size of 50 trials. The test set was composed of the 50 trials per stimulus that immediately followed those used in training. Finally, in an ad hoc analysis, the spectral features implicated in decoding were examined informally to assess the interpretability of the findings.

## 3 Results

### 3.1 Decoding vowels and stimuli from single‐trial FFRs: all trials

**Figure 4:**
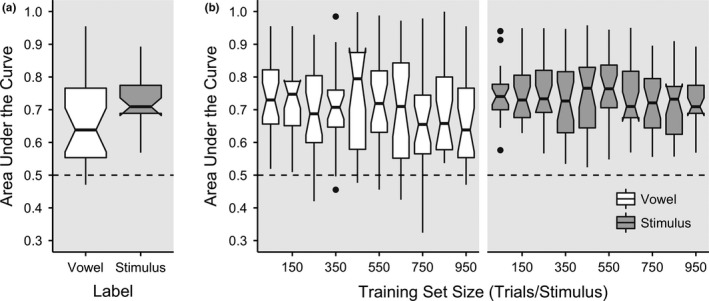
(a) Area under the curve (AUC) measures are displayed for vowel (mean = 0.67; *SD* = 0.15; median = 0.67) and stimulus (mean = 0.73; *SD* = 0.09; median = 0.71) decoding. In this box plot, the dark centerlines correspond to the median, while the top and bottom edges of the boxes correspond to the 25th and 75th percentiles across the 25 participants, respectively. Note that stimulus decoding AUC is averaged across individual one‐versus‐all AUC calculated from each of the four stimuli, the chance level therefore corresponding to 0.50 rather than 0.25. (b) Vowel and stimulus decoding AUC across different sizes of the training set. The *x*‐axis corresponds to the trials per each of the four stimuli (from 50 to 950; step size of 50 trials) that were included as a part of the subset included in training of the classifier. Note that the test set always consisted of the 50 trials per stimulus that immediately followed the training set

Across 25 participants, vowel decoding yielded a mean AUC measure of 0.668 (*SD* = 0.145) with the median of 0.638 (Figure 4a). A one sample *t* test revealed that these AUC values significantly differed from the chance level of 0.5, *t*(24) = 5.798, *p *<* *.0001, 95% CI [0.608, 0.728]. Only two participants exhibited AUC values that were equal to or less than 0.5. Stimulus decoding yielded a mean AUC of 0.729 (*SD* = 0.087) with the median of 0.709 (Figure 4a). A one sample t‐test revealed that these AUC values significantly differed from the chance level of 0.5, *t*(24) = 13.169, *p *<* *.0001, 95% CI [0.693, 0.765]. In all participants, AUC values were higher than 0.5.

### 3.2 Decoding vowels and stimuli from varying sizes of the training set

The distribution of AUC values for vowel and stimulus decoding across training set sizes are shown in Figure 4b. Linear regression analysis was conducted separately for vowel and stimulus to assess decoding performance across all attempts, as well as to assess the effect of the size of training sets on the reliability of decoding. In each case, the dependent variable was logit‐transformed AUC (i.e., 1 and 0 mapped onto ±∞, respectively, with 0.5 mapped onto 0) for a given participant, and the fixed effect was the training set size per stimulus (50, 100, 150, …, and 950). For vowel decoding, the intercept, which modeled the logit‐transformed AUC with the smallest training size, was higher than 0, *b *=* *1.172, *SE* = .157, *t *=* *7.589, *p *<* *.0001, suggesting that decoding performance was higher than chance. There was no effect of training set size, *b *=* *−0.0002, *SE* = .000207, *t *=* *−1.033, *p *=* *.303. For stimulus decoding, the intercept was higher than 0, *b *=* *1.217, *SE* = .104, *t *=* *11.759, *p *<* *.0001, suggesting higher‐than‐chance performance. There was no effect of training set size, *b *=* *−0.000150, *SE *= .000102, *t *=* *−1.475, *p *=* *.142.

### 3.3 Feature interpretability

**Figure 5:**
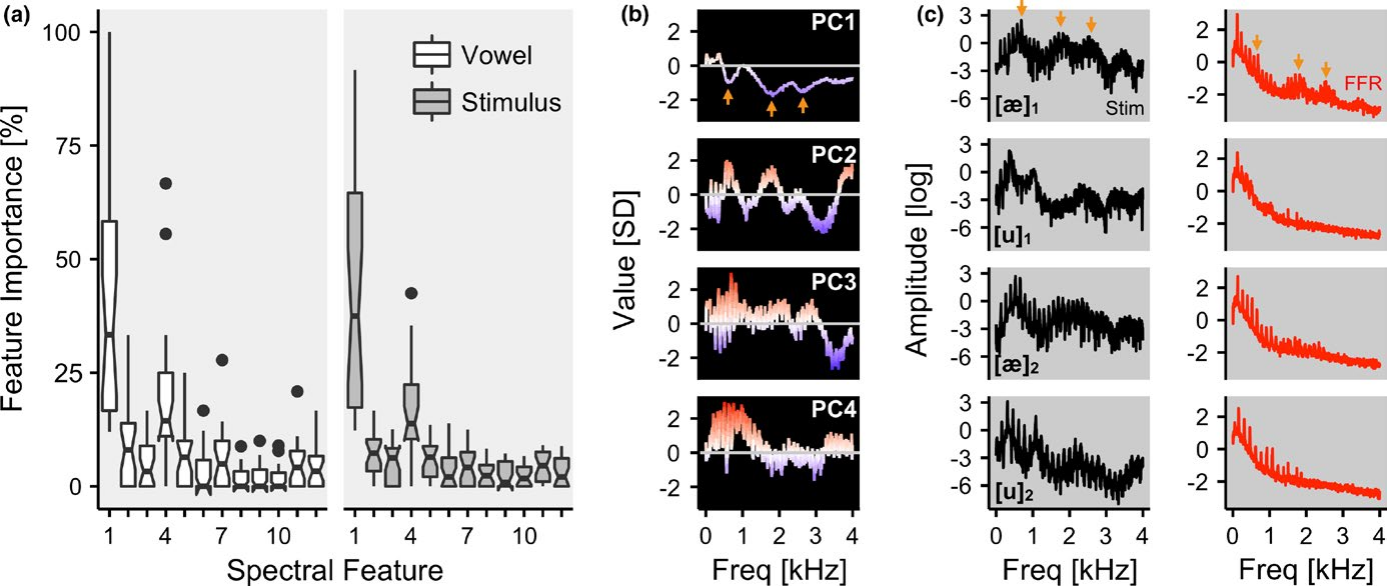
(a) Importance of spectral features during vowel and stimulus decoding (950‐trial training set). The *x*‐axis corresponds to the 12 principal components (PCs) that were used as input features for the classifier. The *y*‐axis corresponds to the percentage of times in which the feature was used by a given decision tree. (b) The top four PCs in the frequency domain. In PC1, which was disproportionately used by the classifiers, three extrema are readily identifiable (arrows). (c) Log‐transformed spectra of the original stimuli (left; black lines) and the grand average frequency following response (right; red lines) are displayed. Three formant frequencies are identifiable (arrows), which also correspond to the three extrema of the PC1 marked with arrows in (b)

Finally, we examined the spectral features used during classification of vowels and stimuli. From each participant, the percentage of times in which a given spectral feature was utilized by individual decision trees was calculated. The distribution of these feature weights is displayed in Figure 5a. An informal inspection of the weights suggested that the PC1 was the most reliable feature in decoding vowels (mean weight = 38%; *SD* = 24%, median = 33%) as well as stimuli (mean weight = 42%; *SD* = 25%; median = 38%). In PC1, three extrema were readily identifiable at 602, 1822, and 2594 Hz (Figure 5b). These values corresponded with the first, second, and third formant frequencies for [æ]_1_ (647, 1864, and 2561 Hz) and [æ]_2_ (553, 2140, and 2327 Hz; Figure 5c; Hillenbrand et al., 1995), and consequently, with the maxima in the spectra of the grand average FFR signal evoked by these stimuli (Figure 5c).

## 4 Discussion

We demonstrate an innovative application of machine learning principles to reliably extract vowel information from the single‐trial speech‐evoked FFRs. In our approach, the raw FFR was first projected onto a spectral feature space defined by a multitude of sounds not used in the experiment, contributing to the generalizability of the results. The feature predominantly used to classify the FFR closely corresponded to the spectral profiles of one of the two vowels used in the experiment. Feature interpretability and generalizability support the likelihood that decoding reflects biologically credible aspects of the FFR, rather than reliable yet trivial features. Finally, reliable decoding performance was achieved with a relatively low training set size of 50 trials per stimulus.

The ability to draw better inferences from the FFR data offers significant potential for auditory neuroscience research. The FFR is elicited preattentively and does not rely on participants’ ability to perform tasks successfully. Moreover, compared to other electrophysiological modalities in which decoding of speech‐evoked responses have been examined (e.g., EEG; Hausfeld et al., 2012; ECoG; Pei et al., 2011; Mesgarani et al., 2014), the FFR has a considerable advantage regarding availability, low monetary cost, and quick implementation time (Skoe & Kraus, 2010). As such, it has been extensively used as a biomarker of auditory function, in both research settings (Banai et al., 2007; Chandrasekaran et al., 2009; Cunningham et al., 2001; Hornickel et al., 2009; Johnson et al., 2005; Kraus & Chandrasekaran, 2010; Krishnan et al., 2009; Krizman et al., 2012, 2014; Russo et al., 2008; Skoe & Kraus, 2010; Song et al., 2008; Wong et al., 2007) as well as in clinical settings, such as infant hearing screening (Herrmann, Thornton, & Joseph, 1995; Mason & Herrmann, 1998). The ability to decode single‐trial FFR opens up new avenues for experimentation in auditory neuroscience. For instance, the effect of dynamically modulated attention on the FFR cannot be easily tested using a block‐by‐block averaging approach since attentional effects may fluctuate on a much more rapid timescale. FFRs are sensitive to short‐term regularity in the input stream (Lehmann, Arias, & Schönwiesner, 2016). This may be the reason for somewhat inconclusive results on the effect of attention on the FFR, where some researchers have shown attention effects (Lehmann & Schönwiesner, 2014; Sörqvist, Stenfelt, & Rönnberg, 2012; Strait, Kraus, Parbery‐Clark, & Ashley, 2010), whereas others have not (Varghese et al., 2015). Implementation of single‐trial decoding approaches would allow flexibility in experimental designs, where trial‐by‐trial behavioral performance can be included as a covariate in FFR analysis. Also, some clinical disorders like developmental dyslexia, a neurological disorder that impacts reading and spelling skills, have been characterized by increased neural variability. For example, a prior study showed individuals with dyslexia exhibited less consistent responses when two subaverages were compared to each other (Hornickel, Zecker, Bradlow, & Kraus, 2012). Assessing variability on a trial‐by‐trial basis could yield more robust and stable group differences rather than assessing signal quality from average responses.

We acknowledge the following limitations of this study. First, participants were presented with two vowels produced by two speakers. These four stimuli fulfill the minimum number of types of speech tokens required to assess representation of segmental and indexical information as exhibited in the FFR. Furthermore, the spectral decomposition approach as derived from an independent set of stimuli (in which novel speakers produced the same vowels) hint at the possibility of generalizability of the decoding performance as reported in the results section. However, future studies should further explore FFRs to multiple speakers and multiple speech sounds. Second, the main focus of this study was to demonstrate the possibility of decoding single‐trial FFRs. For this reason, less emphasis was placed on maximizing the decodability using multiple classifiers (e.g., support vector machine) or different parameters. Future studies, in addition to using a more comprehensive set of speech sounds, could serve the field by exploring different types of computational approaches to optimize the decoding ability.

In summary, we present a set of results that demonstrate the feasibility of decoding speech sound information from single‐trial FFRs. The speech‐evoked FFRs were analyzed in a way that addresses two common pitfalls of a machine learning approach: feature interpretability (countered by using decipherable spectra) and generalizability (countered by deriving features from an independent set of stimuli). We suggest that this approach could be further refined to yield inferences of the neurally driven activity of the auditory pathway, which may not have been plausible by analyzing signals averaged across thousands of trials decoding.

## Conflict of Interest

The authors declare no conflict of interest, financial or otherwise.
